# Current status of the certification of long‐term care insurance among individuals with dementia in a Japanese community: The Hisayama Study

**DOI:** 10.1111/pcn.13204

**Published:** 2021-02-17

**Authors:** Tomoyuki Ohara, Daigo Yoshida, Jun Hata, Mao Shibata, Takanori Honda, Yoshihiko Furuta, Naoki Hirabayashi, Takanari Kitazono, Tomohiro Nakao, Toshiharu Ninomiya

**Affiliations:** ^1^ Department of Neuropsychiatry Kyushu University Fukuoka Japan; ^2^ Department of Epidemiology and Public Health Kyushu University Fukuoka Japan; ^3^ Center for Cohort Studies Kyushu University Fukuoka Japan; ^4^ Department of Medicine and Clinical Science Kyushu University Fukuoka Japan; ^5^ Department of Psychosomatic Medicine, Graduate School of Medical Sciences Kyushu University Fukuoka Japan

It is important to understand the actual situation of subjects with dementia and their long‐term care and activities of daily living in communities for considering appropriate local health policies. This study aimed to investigate the latest findings on the certification for long‐term care insurance and the degree of independence in daily living among subjects with dementia in a Japanese community.

In the Hisayama Study, which is a population‐based epidemiological study in Hisayama town, a full‐community survey for dementia has been repeated every 5 or 7 years since 1985.^1^, ^2^ Among 2,340 residents aged ≥65 years in this town, a total of 2,202 residents (1,270 women and 932 men) (participation rate: 94.1%) participated in a screening examination for cognitive impairment and health status in 2017–2018. Among them, 346 subjects (crude prevalence: 15.7%) who were diagnosed as having dementia were included in this study.

We collected information on the certification for long‐term care insurance from the Division of Health and Welfare of Hisayama town with consent from each participant. We divided the subjects into five categories: no certification, requiring support levels 1 to 2, requiring long‐term care level 1, requiring long‐term care levels 2 to 3, and requiring long‐term care levels 4 to 5.^3^ Regarding the degree of independence in daily living,^3^ there were no dementia subjects certified as M. The detailed definitions for each category are shown in Tables S1‐1 and S1‐2.^3^, ^4^ Appendix S1 provides detailed information on the diagnosis of dementia, long‐term care insurance, sociodemographic factors and health status, statistical analysis, and ethical statement.

Among the 346 subjects with dementia, 69.7% obtained a certification for long‐term care insurance and 56.4% were certified as requiring long‐term care of level 1 or higher (Fig. 1a). In addition, 51.5% of subjects were classified as having a degree of independence in daily living of IIa or more (Fig. 1b). Table S2 and S3 show the clinical characteristics of dementia subjects according to the categories of support or long‐term care and the grades of independence in daily living, respectively. The frequencies of living at health care facilities and hospitalization increased with greater support required or long‐term care levels (Fig. S1). Similar trends were observed for the grades of independence in daily living (Fig. S2). In the age‐ and sex‐adjusted analyses, subjects with lower cognitive function, disability, history of stroke, no regular exercise, lower body mass index, or lower muscle mass and strength were significantly more likely to be certified as requiring long‐term care of level 1 or higher and to have a degree of independence in daily living of IIa or more than those without any of these factors (Table S4 and S5).

**Fig. 1:**
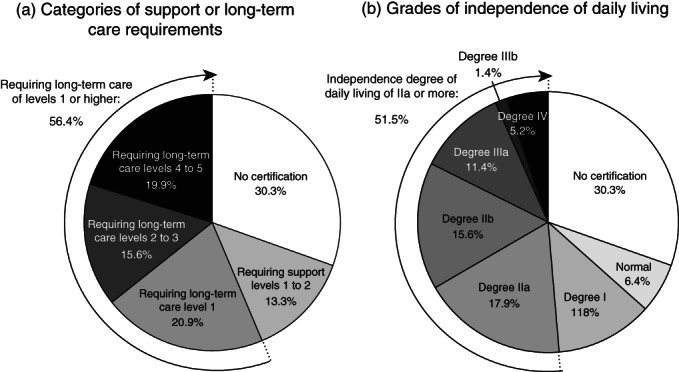
Proportion of subjects certified to receive support or long‐term care (a) and proportion of subjects in each category of independence in daily living (b) among subjects with dementia, 2017–2018. One participant without available data for the grade of independence in daily living was excluded from the analysis.

This cross‐sectional study in a general older Japanese population demonstrated that the crude prevalence of dementia in 2017–2018 was 15.7%, and 69.7% of dementia subjects obtained certification for long‐term care insurance. The GERAS‐J Study, a hospital‐based prospective study in Japan, reported that 70.9% of participants with Alzheimer's disease obtained long‐term care insurance,^5^ which is consistent with our findings. Notably, about one‐third of dementia subjects were not certified for long‐term care insurance in this study. Although these subjects tended to be younger and to have higher cognitive and physical function than the certified subjects (Table S2), our findings highlight the importance of establishing a dementia‐friendly community where dementia subjects are understood, respected, and supported so that even individuals without certification for long‐term care insurance can remain healthy and active in their communities as long as possible.^6^ In addition, our results underscore the importance of conducting detailed surveys in each municipality to identify dementia subjects and to assess their care and support needs.^7^ Furthermore, 6.4% of dementia subjects with lower Barthel index^8^ scores (Table S3) were classified as having a normal grade of independence of daily living. We have no clear explanations for this gap, but this finding may suggest that there are some misclassifications in the evaluation of independence of daily living in this certification system.

Several limitations of this study should be addressed. First, the generalizability of our findings to other regions of Japan and other countries with different lifestyles and social systems is limited. Second, there might be a selection bias caused by the exclusion of residents who did not participate in the initial survey (5.9% of total population). Third, the certification in this town might have been somewhat facilitated by our sharing of the information of subjects without detectable dementia with the family physician and local government members. Further epidemiological surveys will be needed to verify the present findings.

## Disclosure statement

This study was supported in part by a Health and Labour Sciences Research Grant of the Ministry of Health, Labour and Welfare of Japan (20FA1002); by Grants‐in‐Aid for Scientific Research (A) (JP16H02692), (B) (JP19H03863, JP18H02737, and JP17H04126), and (C) (JP20K10503, JP20K11020, JP19K07890, JP18K09412, and JP18K07565), a Grant‐in‐Aid for Early‐Career Scientists (JP18K17925), and a Grant‐in‐Aid for Research Activity Start‐up (JP19K23971) from the Ministry of Education, Culture, Sports, Science and Technology of Japan; and by grants from the Japan Agency for Medical Research and Development (JP20dk0207025, JP20km0405202, and JP20fk0108075). None of the study sponsors had any role in the study design, interpretation of the data, data collection, or drafting of the manuscript. The authors have no conflicts of interest to declare.

## Supporting information


**Figure S1.** Proportion of the places of residence according to the categories of support or long‐term care among subjects with dementia, 2017–2018.

**Figure S2.** Proportion of the places of residence according to the grades of independence in daily living among subjects with dementia, 2017–2018. One participant without available data for the grade of independence in daily living was excluded from the analysis.

**Table S1‐1.** Definition of the certified classification of requiring support or long‐term care in the long‐term care insurance system of Japan.
**Table S1‐2.** Definition of the certified classification of grades of independence of daily living for subjects with dementia in the long‐term care insurance system of Japan.
**Table S2.** Characteristics of the subjects with dementia according to the categories of requiring support or long‐term care level.
**Table S3.** Characteristics of the subjects with dementia according to the grades of independence in daily living.
**Table S4.** Age‐ and sex‐adjusted odds ratios of each factor on the likelihood of being classified as requiring long‐term care of level 1 or higher among subjects with dementia.
**Table S5.** Age‐ and sex‐adjusted odds ratios of each factor on the likelihood of being classified into a grade of daily living of IIa or more among subjects with dementia.

**Appendix S1.** Supplemental methods.
