# Supplementary material for: Current status of the certification of long‐term care insurance among individuals with dementia in a Japanese community: The Hisayama Study
Source: Psychiatry Clin Neurosci. 2021 Feb 17;75(5):182–4. doi: 10.1111/pcn.13204 (PMC8248379; doi:10.1111/pcn.13204)
Supplement: Supplementary file 1 — Figure S1. Proportion of the places of residence according to the categories of support or long‐term care among subjects with dementia, 2017–2018. [file PCN-75-182-s003.pptx]

## Slide 1
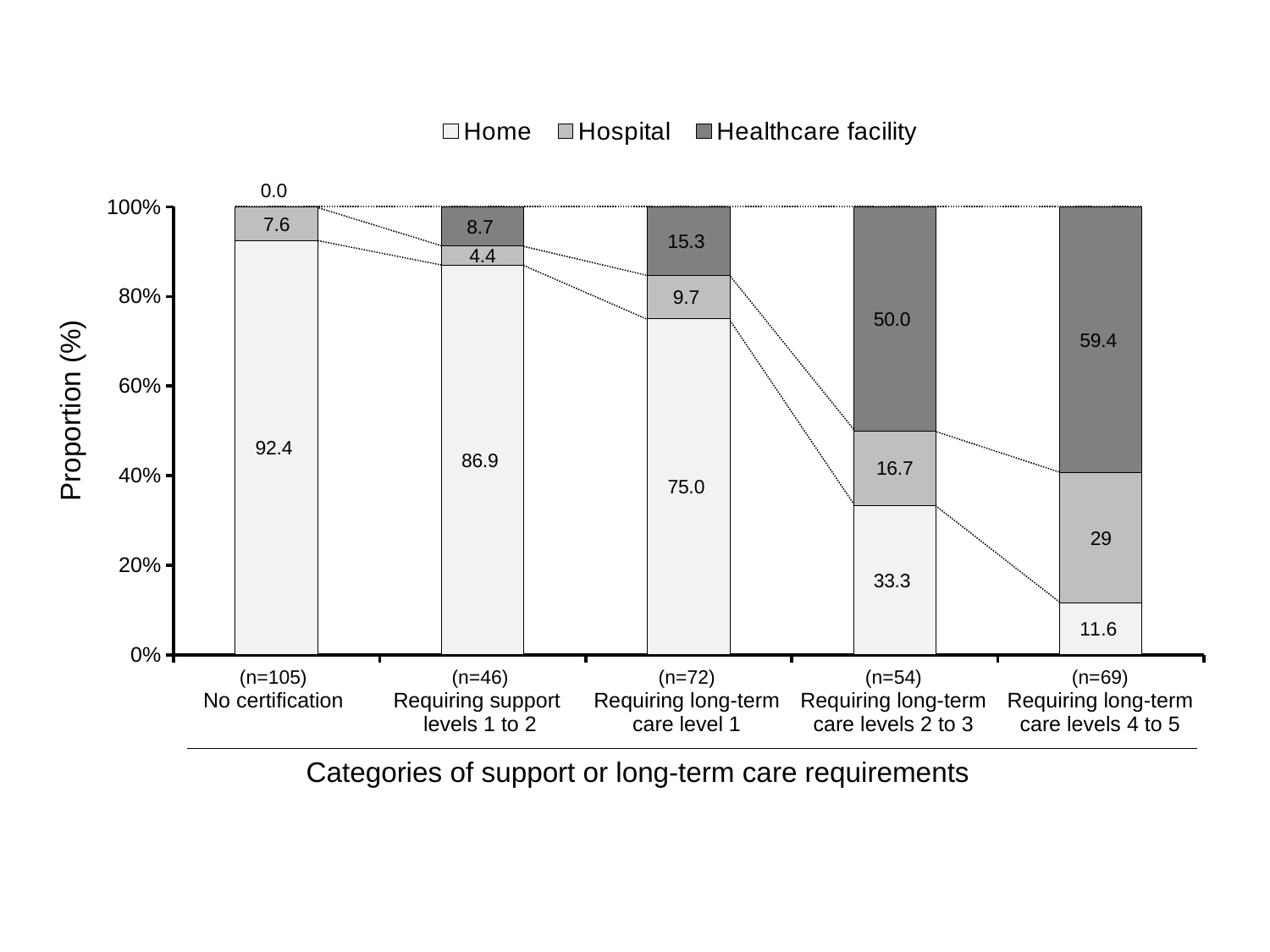

### Chart
| Category | Home | Hospital | Healthcare facility |
|---|---|---|---|
| (n=105)
No application | 92.4 | 7.6 | 0.0 |
| (n=46)
Need support
1&2 | 86.9 | 4.4 | 8.7 |
| (n=72)
Need long-term care
1 | 75.0 | 9.7 | 15.3 |
| (n=54)
Need long-term care 2&3 | 33.3 | 16.7 | 50.0 |
| (n=69)
Need long-term care 4&5 | 11.6 | 29.0 | 59.4 |
Proportion (%)
| (n=105) No certification | (n=46) Requiring support levels 1 to 2 | (n=72) Requiring long-term care level 1 | (n=54) Requiring long-term care levels 2 to 3 | (n=69) Requiring long-term care levels 4 to 5 |
| --- | --- | --- | --- | --- |
Categories of support or long-term care requirements
