# Supplementary material for: Current status of the certification of long‐term care insurance among individuals with dementia in a Japanese community: The Hisayama Study
Source: Psychiatry Clin Neurosci. 2021 Feb 17;75(5):182–4. doi: 10.1111/pcn.13204 (PMC8248379; doi:10.1111/pcn.13204)
Supplement: Supplementary file 2 — Figure S2. Proportion of the places of residence according to the grades of independence in daily living among subjects with dementia, 2017–2018. One participant without available data for the grade of independence in daily living was excluded from the analysis. [file PCN-75-182-s004.pptx]

## Slide 1
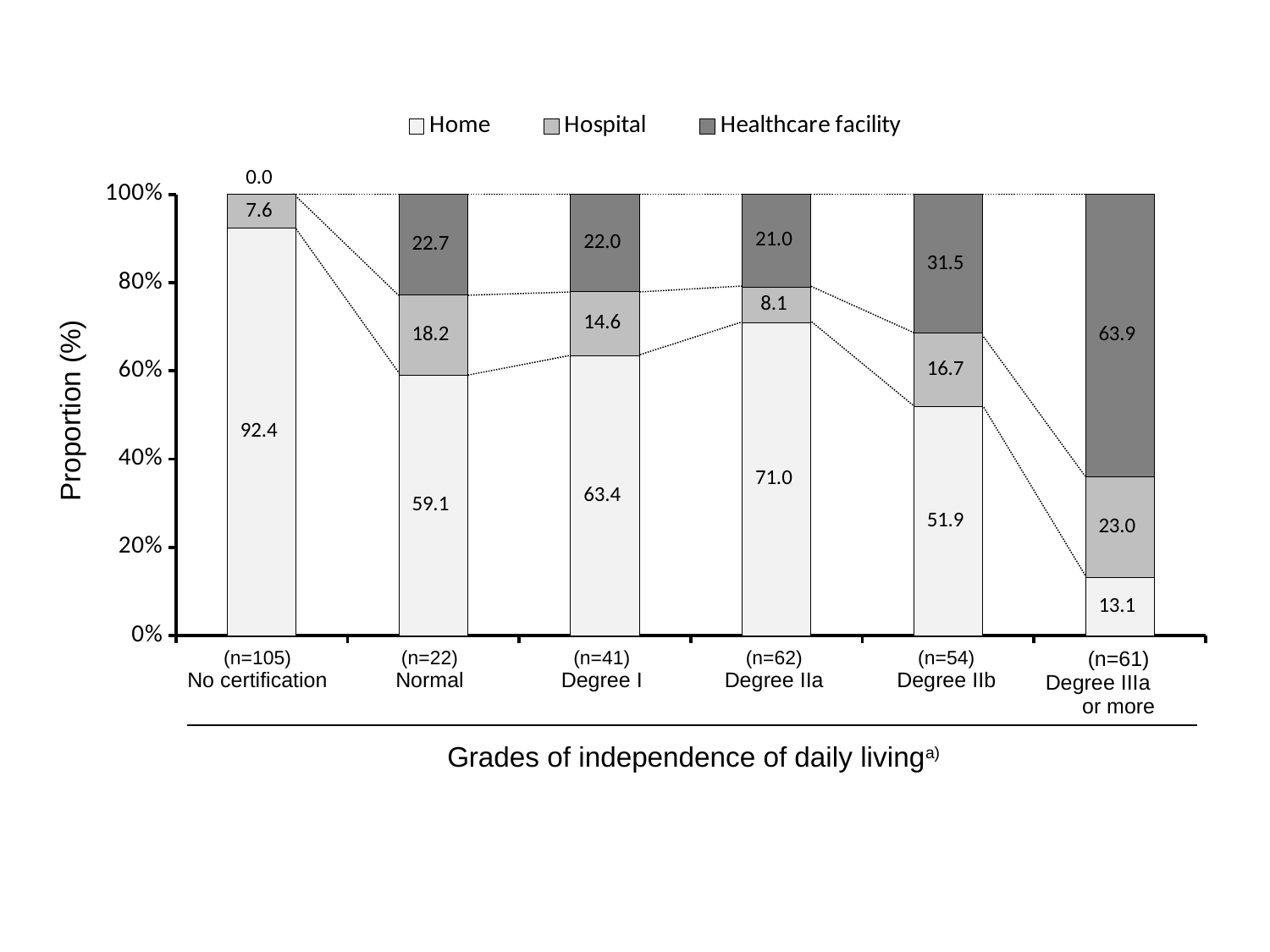

### Chart
| Category | Home | Hospital | Healthcare facility |
|---|---|---|---|
| No | 92.38 | 7.62 | 0.0 |
| Independence | 59.09 | 18.18 | 22.73 |
| 1 | 63.41 | 14.63 | 21.95 |
| 2a | 70.96 | 8.06 | 20.97 |
| 2b | 51.85 | 16.67 | 31.48 |
| 3a | 13.12 | 22.95 | 63.93 |
Proportion (%)
| (n=105) No certification | (n=22) Normal | (n=41) Degree I | (n=62) Degree IIa | (n=54) Degree IIb | (n=61) Degree IIIa or more |
| --- | --- | --- | --- | --- | --- |
Grades of independence of daily livinga)
