# Supplementary material for: Current status of the certification of long‐term care insurance among individuals with dementia in a Japanese community: The Hisayama Study
Source: Psychiatry Clin Neurosci. 2021 Feb 17;75(5):182–4. doi: 10.1111/pcn.13204 (PMC8248379; doi:10.1111/pcn.13204)
Supplement: Supplementary file 3 — Table S1‐1. Definition of the certified classification of requiring support or long‐term care in the long‐term care insurance system of Japan. Table S1‐2. Definition of the certified classification of grades of independence of daily living for subjects with dementia in the long‐term care insurance system of Japan. Table S2. Characteristics of the subjects with dementia according to the categories of requiring support or long‐term care level. Table S3. Characteristics of the subjects with dementia according to the grades of independence in daily living. Table S4. Age‐ and sex‐adjusted odds ratios of each factor on the likelihood of being classified as requiring long‐term care of level 1 or higher among subjects with dementia. Table S5. Age‐ and sex‐adjusted odds ratios of each factor on the likelihood of being classified into a grade of daily living of IIa or more among subjects with dementia. [file PCN-75-182-s002.docx]

**Table S1-1. Definition of the certified classification of requiring support or long-term care level on the long-term care insurance system of Japan**

| **Classification of requiring support or long-term care level** | **Status** |
| --- | --- |
| Requiring support level 1 | Performs daily activities more or less normally.  Requires partial support to prevent the progression of long-term care levels. |
| Requiring support level 2 | Requires partial support to perform activities of daily living. However, activities of daily living could be improved by using any facility services. |
| Requiring long-term care level 1 | Has some difficulties in performing activities of daily living.  Requires partial support to perform activities of daily living. |
| Requiring long-term care level 2 | Has some difficulties in performing activities of daily living.  Requires partial or total support to perform activities of daily living. |
| Requiring long-term care level 3 | Has severe difficulties in performing activities of daily living.  Requires total support to perform activities of daily living. |
| Requiring long-term care level 4 | Has severe difficulties in performing activities of daily living and some difficulties in communication.  Requires total and constant support to perform activities of daily living. |
| Requiring long-term care level 5 | Has severe difficulties in performing activities of daily living and communication.  Requires total and constant support to perform activities of daily living. |

**Table S1-2. Definition of the certified classification of grades of independence of daily living for subjects with dementia on the long-term care insurance system of Japan**

| **Grades of independence of daily living** | **Status** |
| --- | --- |
| Normal | Has no dementia symptoms and performs daily activities more or less normally in the house. |
| Degree I | Has some dementia symptoms, but performs daily activities more or less normally in the house. |
| Degree IIa | Has some difficulties in performing activities of daily living or communication due to dementia out of the house, but can perform daily activities more or less normally with partial support. |
| Degree IIb | Has some difficulties in performing activities of daily living or communication due to dementia in the house, but can perform daily activities more or less normally with partial support. |
| Degree IIIa | Has some difficulties in performing activities of daily living or communication due to dementia in the daytime, but can perform daily activities with total support. |
| Degree IIIb | Has some difficulties in performing activities of daily living or communication due to dementia even at night, but can perform daily activities with total support. |
| Degree IV | Has severe difficulties in performing activities of daily living and communication.  Requires total and constant support to perform activities of daily living. |
| Degree M | Has severe behavioral and psychological symptoms of dementia and/or physical disorder, and requires medical intervention. |

**Table S2. Characteristics of the subjects with dementia according to the categories of requiring support or long-term care level**

|  | Certified support or long-term care level | | | | | |
| --- | --- | --- | --- | --- | --- | --- |
|  | No certification | Requiring support levels 1 to 2 | Requiring long-term care level 1 | Requiring long-term care levels 2 to 3 | Requiring long-term care levels 4 to 5 | No. of missing values |
|  | (n=105) | (n=46) | (n=72) | (n=54) | (n=69) |  |
| ***Demographic and socioeconomic factors*** |  |  |  |  |  |  |
| Age, mean (SD), years | 84 (7) | 87 (5)* | 85 (7) | 86 (7) | 87 (8)* | 0 |
| Female, % | 60.0 | 69.6 | 66.7 | 79.6* | 78.3* | 0 |
| Education ≤ 9 years, % | 47.1 | 68.8* | 51.9 | 46.7 | 61.5 | 145 |
| Have little interaction with others, % | 21.3 | 18.8 | 26.0 | 31.6 | 55.0* | 136 |
| Unemployed, % | 88.8 | 97.1 | 100 | 100 | 100 | 92 |
| Unmarried or without spouse, % | 41.0 | 51.9 | 47.7 | 64.7 | 45.5 | 152 |
| ***Lifestyles*** |  |  |  |  |  |  |
| Habitual alcohol use (current and previous), % | 58.6 | 51.3 | 50.0 | 40.4* | 36.8** | 36 |
| Smoking habits (current and previous), % | 34.0 | 33.3 | 41.2 | 25.5 | 22.8 | 38 |
| Regular exercise, % | 29.9 | 20.0 | 11.8* | 9.8* | 0* | 35 |
| ***Physical functions, examinations and comorbidities*** |  |  |  |  |  |  |
| Barthel index, median (Interquartile range) | 100 (95-100) | 88 (70-100)* | 90 (75-95)* | 50 (40-68)* | 5 (0-25)* | 52 |
| Body mass index, mean (SD), kg/m^2^ | 23.4 (0.7) | 24.0 (0.9) | 22.4 (0.8) | 23.0 (0.9) | 21.4 (0.8)* | 39 |
| Lean mass index, mean (SD), kg/m^2^ | 16.6 (1.9) | 16.9 (1.8) | 15.6 (1.4) | 14.8 (1.5) | 13.1 (1.3) | 243 |
| Hand grip strength, mean (SD), kg | 22.2 (1.4) | 20.5 (2.2) | 19.3 (1.9)* | 17.9 (3.7)* | 9.3 (4.9)* | 199 |
| Knee extension strength, mean (SD), kg | 22.7 (1.7) | 23.6 (3.4) | 19.9 (2.4) | 19.1 (8.7) | n.a. | 232 |
| Systolic blood pressure, mean (SD), mmHg | 152 (4) | 151 (6) | 147 (5) | 151 (5) | 142 (5)* | 33 |
| Diastolic blood pressure, mean (SD), mmHg | 80 (2) | 80 (3) | 78 (3) | 83 (3) | 77 (3) | 33 |
| Use of antihypertensive medication, % | 69.4 | 92.5* | 70.6 | 74.5 | 59.3 | 33 |
| Diabetes mellitus, % | 27.3 | 35.9 | 25.0 | 22.9 | 25.4 | 33 |
| Serum total cholesterol, mean (SD), mmol/L | 5.6 (0.2) | 5.5 (0.3) | 5.3 (0.2) | 5.3 (0.2) | 5.1 (0.2)* | 40 |
| Use of lipid-modifying medication, % | 47.5 | 47.5 | 42.7 | 35.3 | 26.7* | 33 |
| Electrocardiogram abnormality, % | 32.2 | 25.0 | 35.8 | 35.4 | 32.8 | 40 |
| History of stroke, % | 8.7 | 22.6* | 18.9 | 15.4 | 42.1* | 106 |
| History of cancer, % | 21.7 | 16.1 | 18.9 | 7.7 | 10.5 | 106 |
| History of respiratory disease, % | 10.9 | 16.1 | 17.0 | 7.7 | 23.7 | 106 |
| ***Mental status*** |  |  |  |  |  |  |
| Depressive symptom, % | 23.5 | 50.0* | 32.7 | 28.6 | 40.0 | 155 |
| MMSE, median (Interquartile range) | 20 (17-23) | 19 (16-22)* | 15 (12-19)* | 11 (5-15)* | 0 (0-9)* | 116 |

Abbreviations: SD, standard deviation; MMSE, Mini-Mental State Examination; n.a., not assessed.

Regular exercise was defined as engaging in any form of physical exercise at least three times per week during leisure time.

Electrocardiogram abnormalities were defined as Minnesota Code 3-1, 4-1, 4-2, 4-3, or 8-3.

Depressive symptoms were defined as a geriatric depression score ≥6; * p<0.05, ** p<0.01 vs. no certification

**Table S3. Characteristics of subjects with dementia according to the grades of independence in daily living**

|  | The certified independence degree of daily living | | | | | | |
| --- | --- | --- | --- | --- | --- | --- | --- |
|  | No certification | Normal | I | IIa | IIb | IIIa or more | No. of missing values |
|  | (n=105) | (n=22) | (n=41) | (n=62) | (n=54) | (n=61) |  |
| ***Demographic and socioeconomic factors*** |  |  |  |  |  |  |  |
| Age, mean (SD), years | 84 (7) | 84 (9) | 87 (5)* | 86 (6)* | 86 (6) | 87 (7)** | 0 |
| Female, % | 60.0 | 68.2 | 73.2 | 67.7 | 74.1 | 80.3** | 0 |
| Education ≤ 9 years, % | 47.1 | 75.0 | 59.1 | 52.1 | 55.6 | 53.9 | 145 |
| Have little interaction with others, % | 21.3 | 36.4 | 36.4 | 23.8 | 25.9 | 38.9 | 136 |
| Unemployed, % | 88.8 | 100 | 100 | 100 | 98.1 | 100 | 92 |
| Unmarried or without spouse, % | 41.0 | 33.3 | 54.6 | 58.9 | 40.9 | 47.1 | 152 |
| ***Lifestyles*** |  |  |  |  |  |  |  |
| Habitual alcohol use (current and previous), % | 58.6 | 56.3 | 44.7 | 49.2 | 46.7 | 34.6** | 36 |
| Smoking habits (current and previous), % | 34.0 | 43.8 | 26.3 | 42.3 | 28.9 | 21.2 | 38 |
| Regular exercise, % | 29.9 | 5.9 | 15.8* | 16.9 | 4.4** | 3.8** | 35 |
| ***Physical functions, examinations and comorbidities*** |  |  |  |  |  |  |  |
| Barthel index, median (Interquartile range) | 100 (95-100) | 53 (35-85)** | 65 (50-88)** | 85 (75-100)** | 75 (50-95)** | 5 (0-40)** | 52 |
| Body mass index, mean (SD), kg/m^2^ | 23.4 (0.7) | 23.2 (1.2) | 23.2 (0.9) | 22.9 (0.8) | 22.2 (0.9) | 21.9 (0.9)* | 39 |
| Lean mass index, mean (SD), kg/m^2^ | 16.6 (1.9) | 17.1 (0.7) | 16.7 (2.2)* | 15.8 (1.7) | 15.5 (1.2) | 14.8 (1.3) | 243 |
| Hand grip strength, mean (SD), kg | 22.2 (1.4) | 18.8 (4.2) | 19.0 (2.7) | 20.1 (2.1) | 19.5 (2.5) | 13.6 (6.4)* | 199 |
| Knee extension strength, mean (SD), kg | 22.7 (1.7) | 24.6 (6.9) | 20.5 (4.4) | 20.6 (2.7) | 21.2 (3.8) | 18.5 (12.9) | 232 |
| Systolic blood pressure, mean (SD), mmHg | 152 (4) | 155 (7) | 143 (6)* | 150 (5) | 147 (6) | 148 (5) | 33 |
| Diastolic blood pressure, mean (SD), mmHg | 80 (2) | 78 (4) | 77 (3) | 80 (3) | 79 (3) | 82 (3) | 33 |
| Use of antihypertensive medication, % | 69.4 | 76.5 | 81.6 | 81.4 | 73.3 | 55.6 | 33 |
| Diabetes mellitus, % | 27.3 | 35.3 | 34.2 | 28.8 | 24.4 | 18.5 | 33 |
| Serum total cholesterol, mean (SD), mmol/L | 5.6 (0.2) | 4.9 (0.3)* | 5.3 (0.3) | 5.4 (0.2) | 5.2 (0.3)* | 5.3 (0.2) | 40 |
| Use of lipid-modifying medication, % | 47.5 | 23.5 | 55.3 | 42.4 | 35.6 | 29.6* | 33 |
| Electrocardiogram abnormality, % | 32.2 | 18.8 | 21.6 | 33.9 | 36.4 | 41.5 | 40 |
| History of stroke, % | 8.7 | 21.4 | 29.2* | 15.2 | 25.8* | 34.4** | 106 |
| History of cancer, % | 21.7 | 14.3 | 16.7 | 28.3 | 0 | 6.3 | 106 |
| History of respiratory disease, % | 10.9 | 21.4 | 16.7 | 15.2 | 16.1 | 18.8 | 106 |
| ***Mental status*** |  |  |  |  |  |  |  |
| Depressive symptom, % | 23.5 | 37.5 | 61.9** | 31.6 | 25.0 | 30.0 | 155 |
| MMSE, median (Interquartile range) | 20 (17-23) | 18 (13-22)* | 18 (14-22)** | 17 (12-20)** | 12 (7-15)** | 0 (0-8)** | 116 |

Abbreviations: SD, standard deviation; MMSE, Mini-Mental State Examination

Regular exercise was defined as engaging in any form of physical exercise at least three times per week during leisure time.

Electrocardiogram abnormalities were defined as Minnesota Code 3-1, 4-1, 4-2, 4-3, or 8-3.

Depressive symptoms were defined as a geriatric depression score ≥6* p<0.05, ** p<0.01 vs. no certification

One participant without available data for the grade of independence in daily living was excluded from the analysis.

**Table S4. Age- and sex-adjusted odds ratios of each factor on the likelihood of being classified as the requiring long-term care of level 1 or higher among subjects with dementia**

| Factors |  | Age- and sex-adjusted OR (95%CI) | P value |
| --- | --- | --- | --- |
| Education ≤ 9 years | (vs. >9 years) | 0.91 (0.51-1.62) | 0.75 |
| Have little interaction with others | (vs. no) | 2.22 (1.16-4.22) | 0.02 |
| Unmarried or without spouse | (vs. married) | 1.11 (0.58-2.13) | 0.75 |
| Habitual alcohol use (current and previous) | (vs. never) | 0.74 (0.44-1.25) | 0.26 |
| Smoking habits (current and previous) | (vs. never) | 1.94 (0.92-4.09) | 0.08 |
| No regular exercise | (vs. yes) | 4.12 (2.07-8.20) | <0.01 |
| Barthel index | (per 5 point decrement) | 1.27 (1.19-1.35) | <0.01 |
| Body mass index | (per 1 kg/m^2^ decrement) | 1.11 (1.03-1.19) | <0.01 |
| Lean mass index | (per 1 kg/m^2^ decrement) | 1.71 (1.20-2.42) | <0.01 |
| Hand grip strength | (per 1 kg decrement) | 1.12 (1.04-1.22) | <0.01 |
| Knee extension strength | (per 1 kg decrement) | 1.08 (0.998-1.16) | 0.057 |
| Systolic blood pressure | (per 1 mmHg increment) | 0.99 (0.98-0.99) | 0.046 |
| Use of antihypertensive medication | (vs. no) | 0.70 (0.42-1.17) | 0.17 |
| Diabetes mellitus | (vs. no) | 0.95 (0.56-1.60) | 0.84 |
| Serum total cholesterol | (per 1 mmol/L increment) | 0.64 (0.49-0.83) | <0.01 |
| Use of lipid-modifying medication | (vs. no) | 0.64 (0.40-1.02) | 0.06 |
| Electrocardiogram abnormality | (vs. no) | 1.30 (0.80-2.12) | 0.30 |
| History of stroke | (vs. no) | 3.23 (1.55-6.72) | <0.01 |
| History of cancer | (vs. no) | 0.65 (0.33-1.30) | 0.23 |
| History of respiratory disease | (vs. no) | 1.62 (0.77-3.41) | 0.21 |
| Depressive symptom | (vs. no) | 1.19 (0.64-2.23) | 0.59 |
| MMSE | (per 1 point decrement) | 1.29 (1.20-1.38) | <0.01 |

Abbreviations: OR, odds ratio; CI, confidence interval; MMSE, Mini-Mental State Examination.

**Table S5. Age- and sex-adjusted odds ratios of each factor on the likelihood of being classified into a grade of daily living of IIa or more among subjects with dementia**

| Factors |  | Age- and sex-adjusted OR (95%CI) | P value |
| --- | --- | --- | --- |
| Education ≤ 9 years | (vs. >9 years) | 0.99 (0.56-1.78) | 0.98 |
| Have little interaction with others | (vs. no) | 1.22 (0.64-2.31) | 0.55 |
| Unmarried or without spouse | (vs. married) | 1.04 (0.54-2.01) | 0.90 |
| Habitual alcohol use (current and previous) | (vs. never) | 0.81 (0.48-1.37) | 0.43 |
| Smoking habits (current and previous) | (vs. never) | 2.02 (0.96-4.25) | 0.07 |
| No regular exercise | (vs. yes) | 2.79 (1.42-5.49) | <0.01 |
| Barthel index | (per 5 point decrement) | 1.12 (1.08-1.16) | <0.01 |
| Body mass index | (per 1 kg/m^2^ decrement) | 1.07 (1.01-1.15) | 0.03 |
| Lean mass index | (per 1 kg/m^2^ decrement) | 1.60 (1.15-2.23) | <0.01 |
| Hand grip strength | (per 1 kg decrement) | 1.09 (1.01-1.18) | 0.02 |
| Knee extension strength | (per 1 kg decrement) | 1.05 (0.98-1.13) | 0.14 |
| Systolic blood pressure | (per 1 mmHg increment) | 0.99 (0.98-1.006) | 0.36 |
| Use of antihypertensive medication | (vs. no) | 0.87 (0.52-1.44) | 0.58 |
| Diabetes mellitus | (vs. no) | 0.90 (0.53-1.53) | 0.70 |
| Serum total cholesterol | (per 1 mmol/L increment) | 0.84 (0.66-1.07) | 0.16 |
| Use of lipid-modifying medication | (vs. no) | 0.68 (0.43-1.09) | 0.11 |
| Electrocardiogram abnormality | (vs. no) | 1.62 (0.99-2.65) | 0.053 |
| History of stroke | (vs. no) | 2.88 (1.40-5.94) | <0.01 |
| History of cancer | (vs. no) | 0.67 (0.33-1.36) | 0.27 |
| History of respiratory disease | (vs. no) | 1.31 (0.62-2.77) | 0.48 |
| Depressive symptom | (vs. no) | 0.96 (0.51-1.81) | 0.89 |
| MMSE | (per 1 point decrement) | 1.21 (1.14-1.28) | <0.01 |

Abbreviations: OR, odds ratio; CI, confidence

One participant without available data for the grade of independence in daily living was excluded from the analysis.
