# Supplementary material for: Current status of the certification of long‐term care insurance among individuals with dementia in a Japanese community: The Hisayama Study
Source: Psychiatry Clin Neurosci. 2021 Feb 17;75(5):182–4. doi: 10.1111/pcn.13204 (PMC8248379; doi:10.1111/pcn.13204)
Supplement: Supplementary file 4 — Appendix S1. Supplemental methods. [file PCN-75-182-s001.docx]

**Supplemental methods (Appendix S1)**

**Title: Current status of the certification of long-term care insurance among individuals with dementia in a Japanese community: the Hisayama Study**

**Authors:**

Tomoyuki Ohara, M.D., Ph.D.^1,2^, Daigo Yoshida, Ph.D.^2^, Jun Hata, M.D., Ph.D.^2,3,4^, Mao Shibata, M.D., Ph.D.^2,3,5^, Takanori Honda, Ph.D.^2^, Yoshihiko Furuta, M.D., Ph.D.^2,4^, Naoki Hirabayashi, M.D., Ph.D.^2,5^, Takanari Kitazono, M.D., Ph.D.^3,4^, Tomohiro Nakao, M.D., Ph.D.^1^, and Toshiharu Ninomiya, M.D., Ph.D.^2,3^

**Affiliations:**

1. Department of Neuropsychiatry, Graduate School of Medical Sciences, Kyushu University, Fukuoka, Japan.

2. Department of Epidemiology and Public Health, Graduate School of Medical Sciences, Kyushu University, Fukuoka, Japan.

3. Center for Cohort Studies, Graduate School of Medical Sciences, Kyushu University, Fukuoka, Japan.

4. Department of Medicine and Clinical Science, Graduate School of Medical Sciences, Kyushu University, Fukuoka, Japan.

5. Department of Psychosomatic Medicine, Graduate School of Medical Sciences, Kyushu University, Fukuoka, Japan.

**Corresponding Author:**

Tomoyuki Ohara, M.D., Ph.D.

E-mail address: ohara.tomoyuki.287@m.kyushu-u.ac.jp

Department of Neuropsychiatry, Graduate School of Medical Sciences, Kyushu University

3-1-1 Maidashi, Higashi-ku, Fukuoka 812-8582, Japan

Tel: (+81) 92-642-6151

Fax: (+81) 92-642-4854

**Supplemental methods (Appendix S1)**

*Diagnosis of dementia*

In the screening survey of cognitive function, we used the Mini-Mental State Examination (MMSE).^1^ Subjects who met one or more of the following criteria underwent a second screening survey for potential cognitive impairment: (1) MMSE score ≤26 points; (2) a score of ≤4 of a total possible 6 points on the delayed recall test components of the MMSE based on the Modified Mini-Mental State (3MS) Examination^2^ to detect suspected cases of cognitive impairment more sensitively (i.e., 3 questions, each scored 2 points if answered correctly without a hint; 1 point if answered correctly with a hint; and 0 points if answered incorrectly with/without a hint); (3) a failed intersecting pentagon-copying test in the MMSE and/or cube-copying test^3^; and (4) suspected cases based on the manner of speaking and behavior. We conducted secondary comprehensive investigations including the Wechsler Memory Scale of logical memory,^4^ physical and neurological examinations, interviews of the families and attending physicians, and reviews of the medical records by trained psychiatrists as described previously.^5^ Dementia was ascertained using the criteria of the Diagnostic and Statistical Manual of Mental Disorders, Revised Third Edition.^6^ Expert psychiatrists and stroke physicians on the study team adjudicated every case of dementia. During the screening survey, when a participant who was diagnosed as having dementia had some difficulties without any support in the community, we shared this information with his/her family physician and members of the town of Hisayama’s Health and Welfare Office.

*Long-term care insurance*

We collected information on the certification for long-term care insurance of subjects with dementia from the Division of Health and Welfare of the Town of Hisayama with consent from each participant. In the long-term care insurance system in Japan, individuals are classified into seven levels according to the degree of support or long-term care required. These include two support levels (support levels 1 and 2), and five long-term care levels (long-term care levels 1 (disabled) to 5 (severely disabled)).^7^ For purposes of the present study, we divided the subjects requiring support or long-term care into 5 categories: no certification, requiring support levels 1 to 2, requiring long-term care level 1, requiring long term care levels 2 to 3, and requiring long term care levels 4 to 5. In addition, the degree of independence in daily living for subjects with dementia was categorized into the following eight grades according to the standardized physicians’ manual published by the Ministry of Health, Labor and Welfare of Japan: normal, degrees I (impaired), IIa, IIb, IIIa, IIIb, and IV (severely impaired), and M (requiring medical intervention).^8^ In this study, we could not collect one participant’s grades of independence in daily living, and there were no dementia subjects certified as having grade M.

*Measurements of sociodemographic factors and health status*

Each subject completed a self-administered questionnaire that included educational years, marital status, employment status, place of residence (home, healthcare facilities, or hospitalized), frequency of social contact, Barthel Index (an indicator of ADL),^9^ medical treatment (antihypertensive, antidiabetic, lipid-modifying, and antidepressant medications), medical history including stroke, cancer, and respiratory diseases, physical activity, alcohol drinking habits, and smoking habit. A face-to-face interview was conducted by trained registered nurses for all participants, including those who had difficulty completing or who were unable to complete the questionnaire. The Geriatric Depression Scale (GDS)-short version^10^ was used to assess depressive symptoms; prevalent depressive symptoms were defined as a GDS score of ≥6. We used a body composition meter (MC-190; Tanita, Tokyo) to measure body height, body weight, and body fat percentage in light clothing without shoes, and the body mass index (BMI) and lean mass index [(body weight – body weight*body fat percentage) / (body height)^2^] were calculated. Handgrip strength was measured twice for each hand using a Smedley Hand Dynamometer (T.K.K.5401; Takei Scientific Instruments, Niigata, Japan), and knee extension strength was measured twice using a knee extension dynamometer and a strain gauge (T.K.K.5715 and T.K.K.5710e; both Takei Scientific Instruments). For each of these measures, the maximum value was used for the analyses. An automated sphygmomanometer (BP-203 RVIIIB; Omron Healthcare, Kyoto, Japan) was used to measure blood pressure three times in the sitting position after rest for at least ≥5 minutes, and we used the mean of three measurements for the analysis. Diabetes mellitus was defined as follows: fasting glucose level ≥7.0 mmol/L, casual or 2-hour postload glucose levels ≥11.1 mmol/L, hemoglobin A_1c_ ≥6.5%, and/or taking antidiabetic medications. Serum total cholesterol levels were determined enzymatically. Electrocardiogram abnormalities were defined as ST depression (Minnesota Code, 4-1, 2, 3), left ventricular hypertrophy (3-1), or atrial fibrillation (8-3). We classified smoking habits and alcohol habits as habitual (either currently or previously) or not. Regular exercise was defined as adherence to any form of physical exercise ≥3 times a week during leisure time.

*Statistical analysis*

SAS software (version 9.4; SAS Institute, Cary, NC) was used to conduct all of the statistical analyses. The difference in the mean values and the frequencies of the risk factors according to the categories of support or long-term care and the grades of independence in daily living were tested by using an analysis of covariance and a logistic regression analysis, respectively. The logistic regression analysis was used to assess the age- and sex-adjusted associations between each risk factor and the likelihood of being classified into the categories of requiring long-term care of level 1 or higher. In addition, we performed a logistic regression analysis using the degree of independence in daily living of IIa or more as an outcome, because previous clinical studies have reported that individuals with mild or moderate dementia are generally classified as having a degree of independence in daily living of IIa or more.^11,12^ In all analyses, we defined a two-tailed P value of <0.05 as statistically significant.

*Ethical statement*

The Institutional Review Board for Clinical Research of Kyushu University approved the present study. Written informed consent was obtained from the participants.

**References (for the supplemental methods)**

1. Folstein MF, Folstein SE, McHugh PR. "Mini-Mental State": a practical method for grading the cognitive state of patients for the clinician. *J Psychiatr Res* 1975;**12**:189-198.

2. Teng EL, Chui HC. The Modified Mini-Mental State (3MS) Examination. J Clin Psychiatry 1987;**48**:314-318.

3. Maeshima S, Osawa A, Maeshima E, et al. Usefulness of a cube-copying test in outpatients with dementia. *Brain injury* 2004;**18**:889-898.

4. Alzheimer's Disease Neuroimaging Initiative. Procedures manual –Alzheimer's Disease Neuroimaging Initiative.[cited 27 June 2020] Available from https://adni.loni.usc.edu/wp-content/uploads/2008/2007/ADNI_GO_Procedures_Manual_06102011.pdf.

5. Ohara T, Furuta Y, Hirabayashi N, et al. Elevated serum glycated albumin and glycated albumin hemoglobin A_1c_ ratio were associated with hippocampal atrophy in a general elderly population of Japanese: the Hisayama Study. *J Diabetes Investig* 2020;**11**:971-979.

6. American Psychiatric Association. Diagnostic and Statistical Manual of Mental Disorders. 3rd ed, revised. Washington, DC: American Psychiatric Association, 1987.

7. Health and Welfare Bureau for the Elderly, Ministry of Health, Labour and Welfare. The review of long-term care insurance system of Japan (in Japanese). 2020:[cited 27 June 2020] Available from https://www.mhlw.go.jp/content/12300000/000598363.pdf

8. Yoshimura A, Lebowitz A, Bun S, Aiba M, Ikejima C, Asada T. A comparative analysis of dementia inpatient characteristics: results from a nationwide survey of different care facilities in Japan. *Psychogeriatrics* 2016;**16**:34-45.

9. Shah S, Vanclay F, Cooper B. Improving the sensitivity of the Barthel Index for stroke rehabilitation. *J Clin Epidemiol* 1989;**42**:703-709.

10. Sheikh JI YJ. Geriatric Depression Scale (GDS): Recent evidence and development of a shorter version. . *Clin Geron* 1986;**5**:165-173.

11. Tomata Y, Sugiyama K, Kaiho Y, et al. Green tea consumption and the risk of incident dementia in elderly Japanese: the Ohsaki Cohort 2006 Study. *Am J Geriatr Psychiatry* 2016;**24**:881-889.

12. Meguro K, Tanaka N, Kasai M, et al. Prevalence of dementia and dementing diseases in the old-old population in Japan: the Kurihara Project. Implications for Long-Term Care Insurance data. *Psychogeriatrics* 2012;**12**:226-234.
